# Editorial

**Published:** 1902-08-15

**Authors:** 


### EDITORIAL.

In his annual address, the president of one of our State associations, deplores the fact that less than one-fifth of the dentists of the State are members of the Association; and in another part he recommends that the Association spend a part of its accumulated funds in a banquet or some other social function. We also note that the majority of the time of that particular meeting seems to have been taken up in the transaction of routine business and the exchange of complimentary remarks of an entirely personal and somewhat fulsome character. We often wonder how long it will take those who value the assembling of themselves together in dental societies to reach the conclusion that for the rank and file of the profession such things are not of first importance, and that they will not spend money in travel, and lose time which is money, to attend "pink teas," or "thimble parties?" Is it not time to revise the methods of conducting these organizations by delegating the executive functions to a small, but able and willing committee, which can transact the business necessary with dispatch and satisfaction? This will leave the time open for the reading and discussion of papers, giving of clinics and inspection of appliances; any surplus funds should be utilized to the best advantage in securing papers, clinics and exhibits, which will not only attract attention but be of real value to all who witness them. This it seems to us would bring the largest results in the way of attendance and of the most stable character.

